# Means and Methods for Obtunding Dentine

**Published:** 1904-11-15

**Authors:** F. C. Collins

**Affiliations:** Detroit


### MEANS AND METHODS FOR OBTUNDING DENTINE.

BY F. C. COLLINS, D.D.S., DETROIT.

Read before the Michigan Dental Association, June 29, 1904.

It is one of the serious difficulties with which the dentist has to contend that nearly all the tissue upon which he operates is more than ordinarily sensitive. This is particularly true of the dentine whether in health or disease. It is the pain inflicted in excavating cavities that is largely responsible for the fear the patient experiences in approaching the dental chair, and that makes so great a drain on the nervous energy of the operator in his endeavor to overcome.

To devise a speedy, safe and certain means of obtunding dentine has long engaged the attention of the profession, but as yet it remains one of the dental problems that have been only partially solved.

Among the simpler methods used may be mentioned the application of heat and cold, the former by projecting a current of hot air on the exposed surface, the latter by dropping some highly volatile fluid such as alcohol, chloroform or ether into the cavity and allowing evaporation to take place. The depth to which either method obtunds is necessarily very limited and would require frequent repetitions to be of much service.

There are certain drugs, owing to the properties they possess, that have more or less obtunding power. Among these are the escharotics, such as carbolic acid, chromic acid, trichloracetic acid and nitrate of silver. Their action on the exposed ends of the dentinal tubules materially lessens the sensibility of the dentine to pain. A group of drugs possessing astringent properties have also been used. The most common are chloride of zinc, tannic acid and terchloride of gold. Either one combined with alcohol and placed in a cavity by means of a pledget of cotton some time previous to excavating renders the operation much less painful. Some of the essential oils, such as oil of cloves and oil of cajeput, by overstimulating the dentine, act as obtundents. They have not much immediate effect, but placed in the cavity a length of time before operating, the good results produced are marked.

Drugs possessing anesthetic properties are among the most useful for obtunding purposes. Aconite, atropine, camphor, chloral and cocain, are the principal ones used. They have most effect where the dentine is not very compact, and this effect may be increased by means of pressure in applying them.

All the foregoing means of obtunding dentine are but partial in their action, and penetrate to a limited distance. Necessarily, in the excavating of carious cavities, or in trimming a tooth for the reception of a crown, they are of little value. It was only after the introduction of cataphoric appliances that it could be said we had a means of rendering dentine absolutely insensitive to pain.

When these appliances were first brought to the notice of the profession, they were received with high favor, but, in a short time a reaction set in owing to the many failures in practical work. This was due mainly to two causes: In the first place, some of the instruments placed on the market were deficient in some important particular. Secondly, many dentists had little or no practical knowledge of the principles of electricity, and through lack of this did not take all necessary precautions in applying the instrument. They were confused by the claims of rival advocates who championed the respective merits of high and low voltage instruments—of those with or without a milliampere meter attached—of appliances where the patient was in shunt or in series. Unfortunately, the element of cheapness entered in those outfits that had no milliampere meter, and, without that guide, cataphoric medication is little better than guess work. Some deficiency in insulating the tooth would result in absolute failure as far as obtunding the dentine was concerned, or, where insulation was perfect, the current might be so great as to injure the pulp. There can be no doubt that a cataphoric apparatus run by a battery of fairly high voltage arranged so that it may be raised or lowered, with a rheostat to regulate the flow of current and a mill'ameter to measure it, will, in the great majority of cases, give satisfactory results. It matters little whether the patient be in series or in shunt. The time usually required to obtund dentine by cataphoric means will always be an objection, still I look forward to its return to favor, and to a place in the best dental offices.

Within the past few years an apparatus, the invention of a California dentist, has met with considerable favor among the dentists of that State for its success in obtunding sensitive dentine. It acts by directing a spray or small stream of hot water into the cavity under pressure of compressed air. A small appliance is attached to the hand-piece. A rubber tube leads from this to a tank filled with compressed air. Another tube leads to an electric or other heater attached to the fountain spittoon through which a stream of water flows. The spray of heated water strikes the dentine at the point of the bur. It is claimed that this prevents any pain being felt. This method has the advantage over cataphoresis in its effect being almost immediate. It necessitates the office being equipped with a fountain spittoon to carry away by means of the saliva ejector the water that would otherwise collect. The principle in this method seems directly opposite to that used in applying hot air. It may be that in both cases it is heat that acts and not the drying of the dentine as was supposed to be the case with hot air.

A more recent appliance for obtunding dentine has lately been placed on the market. It, also, is the invention of a dentist of the Pacific Coast. In principle it is not new, only in its application. Briefly described, the apparatus consists of a receptacle for holding ether. A rubber tube leads from this to an atomizer attached to the hand-piece. From the atomizer another tube leads to a tank filled with air at about twenty pounds pressure. A pledget of cotton or piece of spunk is first placed in the cavity. Against this the spray is directed. After a short time the pledget is removed and the spray is allowed to enter the cavity unprotected. In from three to ten minutes the dentine is so obtunded that excavation may proceed without pain. It requires a temperature of about 46 degrees Fahrenheit to bring this about. In the use of this apparatus, the only precaution necessary is to have the rubber dam placed over the nose. Those who have used this appliance report no bad after-effects to the pulp. The outfit is simple and comparatively inexpensive. If it works as satisfactorily as its admirers claim, it will prove a valuable aid to the dentist.

From the foregoing it will be seen that the problem of obtunding dentine has been practically solved. It still remains, however, to devise a method of doing the work promptly. Until this is done, the use of obtunding appliances in ordinary dental offices will be limited.

DISCUSSION.

Dr. E. T. Loeffler: The great difficulty with almost all methods of obtunding dentine is that those which are most efficient require so much time for satisfactory results, that busy or impatient dentists use expedients that make the pain bearable for the patient and feel satisfied. This has given patients the idea that painless dentistry is a myth. The essayist has noted that the great fault with cataphoresis, the most efficient obtundent, is the time consumed. The same may also be said of all methods of desiccation. It takes time to dry out the dentine sufficiently by any process to obtain profound insensibility. Not so much time is required in resorting to the escharotics, but these are painful in application. We haven't time to wait for results from good methods. We are too impatient. This is one of the difficult problems and we do not give it the consideration our patients wish we should. It would really be better for us, in a business way, if we should make more painless operations, or at least do what we can to modify the pain in all operations.

Dr. B. H. Lee: I think we should make more use of the chemicals than many of us do. The cocain solution, when properly used, gives very satisfactory results. I now use the ether solution of cocain called "vapo-cocain," with gratifying results. I do not vaporize it. but place it in the cavity without thoroughly drying it and wait five minutes and sometimes re-apply. I have had very good results in this way. Warm carbolic solutions of cocain are also effective.

Dr. C. H. Worboys: While at Ann Arbor last winter attending the porcela'n course, Dr. Runyan, of South Haven, demonstrated a method of obtunding the upper anterior teeth that is unique, and I have used it since on several occasions with very good results. The method consists in placing a pledget of cotton, which had been saturated with a ten percent solution of cocain, in the nostril of the side on which the operation is to be made, or in both nostrils if desirable. The application becomes effective in about five minutes, so that sensitive cavities may be excavated without pain to the patient. I find a three or four percent solution is just as effective as a stronger solution and less risky. No excess should be permitted to drain back into the throat. It is a simple and effective treatment and a number of men have used it with as satisfactory results as myself.

DR. ZEDERBAUM: Some classes of cavities are very difficult to obtund with chemicals. Shallow or three walled cavities are difficult, because you can not produce absorption by pressure. The dentine does not ordinarily absorb, it only transfers. To make the medicines penetrate, they must be forced inward by cataphoresis, pressure, or heat, and it is usually difficult to apply these measures in the cavities where we most desire an obtundent effect. I have had good results from applying a few crystals of menthol to shallow cavities and dissolving this in the cavity with a drop or two of absolute alcohol, and then throwing a steady but small stream of compressed air into the cavity until it is dry. I don't know whether the menthol acts chemically by absorption, or whether it is the physical effect of evaporating these volatile drugs, and in so doing produce an excessive evaporation of the tooth moisture.

DR. C. J. HAND: I have had good results from using the solution of cocain in potassium with pressure. I do not think we should use strong cocain solutions in the nostrils, as it would be easy for an excess to flow back into the throat where it would be dangerous to breathing.

DR. WORBOYS: I do not use over a three or four percent solution and use adrenalin chloride with it, so that the astringent action of the adrenalin localizes the effect. There is no possibility that any can get back into the throat as there is no excess. I have used this method for extirpating pulps with satisfactory results. My method is to saturate the cotton and place it in the nostril and then proceed to put the dam over the teeth and by the time I am ready to begin excavating the tooth, the obtundent action has become effective. It may seem risky and mythical, but you can easily try it on the first troublesome case you get and I believe you will be surprised at the results.

Dr. F. W. Joslin: In using cocain as Dr. Worboys suggests, there is a possibility that we may so thoroughly anesthetize the apical area that we shall lose our sense guide when removing the pulp with the broach. It would be an easy matter for us to protrude the broach in some teeth into the apical space and do much injury, especially should the broach become infected, and be the means of carrying this infection into this area. A troublesome alveolar abscess would surely follow. It will be wise for us when using this or any similar obtundent to avoid the probability of carrying infection from the canal to the apical space.

Dr. L. P. Hall: If patients are willing to pay for the time spent in doing their work by tedious painless methods, we should be willing to allow them this privilege, unless we have so many patients demanding our time that we could not spare the time. The trouble frequently is that the patients who need anesthetic treatment most are least able to pay the price. In these cases we must do our duty if we do lose time.

Dr. Collins: My paper is not exhaustive as the discussion plainly shows. For quick results alone drugs are inefficient, some means of application is essential. But if we can proceed with deliberation there are numerous drugs that can be used with good results. The use of cocain by pressure is now coming into use, and much is probably to come from this agent and process.

